# Comparative Analysis of Cervical Disc Arthroplasty and Anterior Cervical Discectomy and Fusion: Trends, Demographics, and Clinical Outcomes in a Nationwide Inpatient Sample

**DOI:** 10.3390/jcm14186559

**Published:** 2025-09-18

**Authors:** Assil Mahamid, David Maman, Dan Fishman, Marah Hodruj, Hadar Gan-Or, Amit Keren, Saleem Samara, Ali Yassin, Yaron Berkovich, Eyal Behrbalk

**Affiliations:** 1 Department of Orthopedic Surgery, Hillel Yaffe Medical Center, Hadera 3820302, Israel; 2 Rappaport Faculty of Medicine, Technion University Hospital (Israel Institute of Technology), Haifa 3200003, Israel; 3 Department of Orthopedic Surgery, Carmel Medical Center, Haifa 3436212, Israel; 4 Bnai-Zion Medical Center, Haifa 3339419, Israel

**Keywords:** cervical disc arthroplasty, anterior cervical discectomy and fusion, national inpatient sample, big data, NIS

## Abstract

**Introduction:** Cervical disc disease is a common cause of disability worldwide. Two surgical options for refractory CDD are anterior cervical discectomy and fusion (ACDF) and cervical disc arthroplasty (CDA). While ACDF is well established, CDA offers motion preservation and has shown promising outcomes. This study compared utilization trends, patient characteristics, and hospitalization outcomes of ACDF and CDA using a large national dataset. **Methods:** We analyzed patients in the Nationwide Inpatient Sample (2016–2019) undergoing ACDF or CDA, identified using ICD-10 codes. After exclusions, 97,999 patients were included. Propensity score matching yielded 11,415 pairs, enabling balanced comparisons of demographics, comorbidities, complications, length of stay (LOS), and hospital charges. **Results:** CDA utilization increased during the study period. Compared with ACDF, CDA patients were younger and more likely to have private insurance. Following matching, both groups were demographically similar. CDA was associated with a slightly shorter LOS (1.32 vs. 1.39 days) but significantly higher charges (USD 82,431 vs. USD 58,472). In terms of complications, dysphagia was more frequent after ACDF, whereas cervical spinal cord injury and urinary tract infections were slightly more common after CDA, though rare overall. **Conclusions:** CDA is increasingly adopted in younger, privately insured patients and demonstrates comparable safety with ACDF. Its advantages include motion preservation, shorter hospitalization, and lower dysphagia rates, though at the expense of higher costs. These findings support the selective use of CDA as a viable alternative to ACDF in appropriately chosen patients.

## 1. Introduction

Cervical disc diseases (CDD) are a leading cause of disabilities and decreased quality of life [1,2,3] worldwide, with prevalence increasing with age [4,5,6]. The related pathologies are varied and highly common in the population [7,8,9,10,11]. Two widespread surgical interventions for symptomatic CDD patients who have failed or are not suitable for conservative treatment are anterior cervical discectomy and fusion (ACDF) and the newer, motion-preserving, cervical disc arthroplasty (CDA). The ACDF is currently the most commonly performed procedure due to its extended history of use and a larger body of clinical evidence supporting its effectiveness. However, the popularity of CDA has been rising in recent years, and like ACDF, it can be used for both single-level and multi-level diseases.

Three major drawbacks of ACDFs for single-level disease post-surgery are limited neck mobility, the potential for adjacent segment disease, and longer recovery time. These drawbacks can be addressed with the more expensive CDA procedure [12], which has shown long-term results that are not inferior and even superior to those of ACDF [13,14,15,16,17,18]. Previous studies have attempted to characterize the patient population more likely to undergo the CDA procedure over ACDF for single-level disc disease and compare the inpatient outcomes [12]. These studies show shorter lengths of stay with CDA and highlight that younger patients, patients with private insurance, and patients with higher median household incomes are more likely to undergo this procedure [19,20,21,22].

Although previous studies have utilized the NIS database to compare CDA and ACDF, these investigations were conducted over a decade ago, employed the now outdated ICD-9 coding system, and did not account for advancements in CDA technology. In contrast, our study incorporates the updated ICD-10 coding system, includes a significantly more extensive and robust patient cohort, and reflects technological improvements in CDA, providing a more accurate and contemporary comparison. Our study utilizes a comprehensive dataset of 97,999 patients to compare ACDF with CDA. The primary objective is to contribute to the ongoing discourse regarding the efficacy of CDA by elucidating its practical implications, including patient demographics, complications, costs, length of hospital stay, and mortality rates. This investigation aims to provide valuable insights that can guide policymakers and ultimately enhance patient-centered care.

## 2. Methods

### 2.1. Data Source

This investigation employed data from the National Inpatient Sample (NIS), a nationally representative database developed by the Agency for Healthcare Research and Quality (AHRQ) as part of the Healthcare Cost and Utilization Project (HCUP). The NIS is the largest publicly accessible, all-payer inpatient healthcare dataset in the United States, systematically sampling approximately 20% of all hospital discharges from HCUP-affiliated institutions. This sampling framework encompasses roughly 7 million unweighted admissions annually and, when adjusted using the discharge-level sampling weights provided by HCUP, allows for the generation of robust national estimates and comprehensive epidemiological assessments.

For the present study, data spanning 1 January 2016 through 31 December 2019 were analyzed, representing the most recent and complete period available at the time of analysis. Within the NIS, each discharge record—referred to as a “case”—is assigned a statistical weight, with each weighted record corresponding to approximately five actual inpatient encounters nationwide. This methodology enables precise extrapolation to the national inpatient population, thereby enhancing both the external validity and statistical rigor of the study’s findings.

### 2.2. Cohort Definition and Selection Criteria

The National Inpatient Sample (NIS) database was queried for the period 2016–2019 to identify adult patients (aged ≥ 18 years) who underwent single-level ACDF or CDA. Procedural identification was performed using International Classification of Diseases, Tenth Revision (ICD-10) procedure codes specific to these operations, as detailed in the Table 1. The final cohort comprised 97,999 patients, including 85,584 who underwent ACDF and 11,415 who underwent CDA.

Patients with non-elective admissions or those who had undergone surgery prior to the index hospitalization were excluded. In addition, cases with incomplete or inconsistent records—such as missing procedural codes, demographic variables, or other critical data—were removed to preserve the accuracy and reliability of statistical analyses. This exclusion strategy minimized the potential for bias arising from incomplete datasets and ensured methodological rigor.

### 2.3. Outcome Variables (End Points)

Procedural identification was based on the International Classification of Diseases, Tenth Revision, and Procedure Coding System (ICD-10-PCS) codes specific to single-level ACDF and CDA, as detailed in Table 1. Comorbidities were identified through a review of patient-specific ICD-10-CM diagnosis codes.

Primary outcomes included in-hospital mortality, length of stay, total hospitalization costs, and perioperative complication rates. Complications were identified using ICD-10 codes and encompassed dysphagia, postoperative anemia due to blood loss, cervical spinal cord injury, urinary tract infection, acute renal failure, pneumonia, blood transfusion requirement, venous thromboembolism, pulmonary edema, ileus, feeding tube placement, dural tear, sepsis, pulmonary embolism, and mortality. These definitions were applied consistently across both groups to ensure methodological uniformity and comparability of results.

### 2.4. Statistical Analysis

All statistical analyses were conducted using SPSS version 26 (IBM Corp., Armonk, NY, USA) and MATLAB 2024 (MathWorks, Natick, MA, USA). Categorical variables were compared using Pearson’s χ^2^ test, and continuous variables were evaluated using independent-sample t-tests. A two-tailed *p*-value < 0.05 was considered statistically significant.

To minimize selection bias and control for confounding inherent in observational studies, propensity score matching (PSM) was employed. This method facilitated the creation of statistically comparable cohorts of patients undergoing ACDF or CDA by matching individuals on key demographic, hospital-related, and clinical characteristics. PSM enhances the validity of causal inferences by approximating the balance achieved in randomized controlled trials, thereby improving the robustness and reliability of comparative analyses.

Propensity scores were estimated using a multivariable logistic regression model incorporating 34 covariates spanning three domains: (1) Hospital characteristics—hospital size, location (urban vs. rural), teaching status, geographic region, and total annual discharges. (2) Demographic and socioeconomic factors—patient location (urban vs. rural classification), median household income quartile, race, age, and primary payer status (Medicare, Medicaid, private insurance, self-pay, or other). (3) Preoperative comorbidities—24 conditions including hypertension, dyslipidemia, obstructive sleep apnea, chronic anemia, alcohol abuse, osteoporosis, neurodegenerative disorders (Parkinson’s disease, Alzheimer’s disease, and dementia), chronic kidney disease, congestive heart failure, chronic lung disease, diabetes mellitus, inflammatory bowel disease, liver disease, obesity, fibromyalgia, thyroid disorders, prior myocardial infarction, peripheral vascular disease, prior cerebrovascular accident, any neoplasm, neoplasms of lymphoid and hematopoietic tissue, and any other recorded preoperative health condition.

Matching was performed using MATLAB, yielding two final cohorts of 11,415 patients each with comparable baseline characteristics. Matching criteria included hospital size, patient location (urban–rural classification), median household income quartile, hospital region, comorbidity profile, and total number of hospital discharges within the NIS dataset.

### 2.5. Ethical Consideration

This study received exempt status from the Institutional Review Board (IRB) owing to the fully de-identified nature of the National Inpatient Sample (NIS) dataset, in accordance with ethical standards for research involving human subjects.

## 3. Results

**Figure 1:**
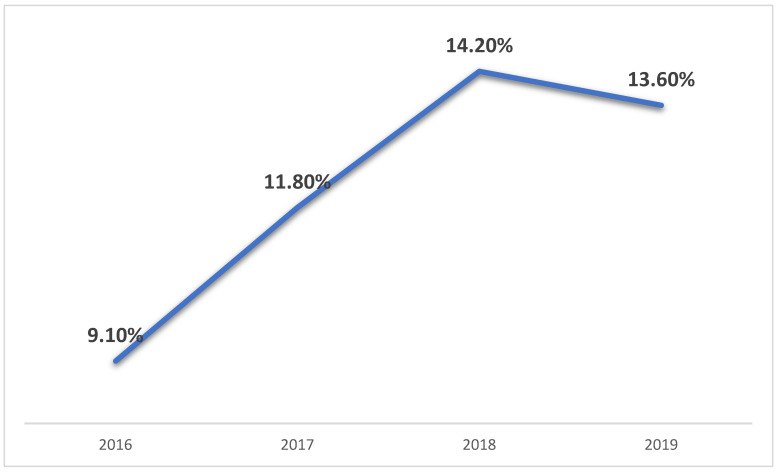
Annual proportion of cervical disc replacement surgeries relative to total disc arthroplasty and ACDF procedures (2016–2019).

Over the past few years, CDA has been used more often compared to ACDF, as shown in Figure 1. From 2016 to 2019, the share of CDA among all CDA and ACDF procedures steadily increased, with the trend reaching statistical significance (*p* = 0.001). This rise reflects a growing preference for CDA in suitable patients, likely influenced by advances in technology, supportive clinical evidence, and increasing surgeon experience with the procedure.

Among the 85,584 patients undergoing ACDF and the 11,415 patients undergoing CDA, the most clinically relevant difference was age. ACDF patients were, on average, substantially older than CDA patients (55.6 vs. 47.2 years, *p* < 0.001), which translated into distinct payer distributions. Medicare was the primary expected payer for one-third of ACDF patients (33.9%) compared to only 10.7% of CDA patients, whereas private insurance predominated among CDA patients (64.9% vs. 44.5%). These differences reflect the contrasting patient populations typically selected for each procedure. In contrast, sex distribution was similar between groups (approximately 52% female), and, therefore, not clinically meaningful despite reaching statistical significance (Table 2).

In Table 3, patients undergoing ACDF exhibited a substantially higher comorbidity burden compared to those undergoing CDA. The most notable differences included hypertension (43.7% vs. 25.1%), dyslipidemia (30.0% vs. 17.3%), and diabetes mellitus (19.5% vs. 9.6%), all of which are common and clinically relevant chronic conditions that can affect perioperative risk and long-term outcomes. Additionally, chronic lung disease (8.0% vs. 3.2%) and chronic kidney disease (3.8% vs. 1.1%) were more frequent among ACDF patients, further reflecting a sicker patient population. These findings align with the older mean age of the ACDF cohort and suggest that CDA candidates are generally younger and healthier, consistent with the more selective surgical indications for arthroplasty.

To address potential selection bias and baseline differences, a propensity score matching (PSM) analysis was performed, yielding 11,415 patients in each cohort. This process achieved an excellent balance across demographic and clinical characteristics, effectively minimizing confounding. After matching, the cohorts were nearly identical in age (47.3 vs. 47.2 years), sex distribution (~52% female), insurance status, and the prevalence of common comorbidities such as hypertension and dyslipidemia. The absence of clinically or statistically significant differences confirms the robustness of the matching process and supports the validity of subsequent outcome comparisons (Table 4).

Following propensity score matching, both ACDF and CDA were associated with short postoperative hospitalizations. Although CDA patients had a slightly shorter mean length of stay compared with ACDF (1.32 vs. 1.39 days), this difference is unlikely to be clinically meaningful. In contrast, a substantial difference was observed in hospital charges: CDA was associated with markedly higher costs than ACDF (USD 82,431 vs. USD 58,472). This disparity likely reflects the higher expense of implant technology and instrumentation used in arthroplasty. These findings highlight that while perioperative recovery is similar between procedures, CDA imposes a greater economic burden, underscoring the importance of balancing clinical outcomes with cost considerations in surgical decision-making (Table 5).

After propensity score matching, complication rates remained low for both procedures. Dysphagia was the most notable difference, occurring more frequently following ACDF than CDA (4.9% vs. 3.6%), consistent with the greater esophageal retraction required in fusion procedures. Other complications were rare in both cohorts and, although some reached statistical significance, their absolute incidence was extremely low (<0.5%) and of limited clinical impact. These included slightly higher rates of spinal cord injury and urinary tract infection in the CDA group, and isolated increases in transfusion and venous thromboembolism in the ACDF group. Collectively, these findings indicate that both procedures are generally safe, with dysphagia representing the most clinically relevant difference in complication profile (Table 6).

## 4. Discussion

ACDF is a well-established surgical intervention for patients with severe or refractory cervical spine pathology who have not achieved symptomatic relief through conservative management [23]. CDA was developed as a motion-preserving alternative designed to maintain cervical spine biomechanics, reduce stress transfer to adjacent segments, and thereby mitigate the risk of ASD [24,25]. Despite promising biomechanical and clinical rationale, the question of whether CDA offers superior long-term outcomes compared with ACDF remains the subject of ongoing debate [26].

This study leveraged a large, propensity score–matched cohort from the National Inpatient Sample (NIS) to evaluate the epidemiological trends and complication profiles of ACDF versus CDA. By incorporating updated ICD-10 coding, a broader and more contemporary timeframe (2016–2019), and improved propensity score matching methodology, our analysis provides a more precise and comprehensive assessment than previous large-scale studies. Our findings demonstrate a marked increase in CDA utilization over the past decade, consistent with previously published reports. One study [27] observed an increase in CDA procedures from 4.0% to 14.2% between 2010 and 2018, followed by a plateau from 2018 to 2021. Similarly, Singh BS et al. [28] documented a 25.25% rise in ACDF procedures from 2011 to 2014 and an extraordinary 654.24% increase in CDA procedures from 2011 to 2019, with subsequent stabilization in the rates of both interventions. The growing adoption of CDA over ACDF is likely influenced by earlier evidence suggesting superior postoperative functional mobility with arthroplasty, potentially mitigating biomechanical stress on adjacent segments and lowering the incidence of adjacent-segment degeneration [29,30].

In our cohort, patients undergoing CDA were generally younger and demonstrated fewer comorbidities. CDA is frequently selected for younger individuals with preserved baseline segmental motion and without advanced degenerative changes of the cervical spine, as it offers the potential for greater postoperative mobility and segmental flexibility compared with ACDF [12]. The procedure’s success relies on the structural integrity of adjacent facet joints and spinal ligaments to maintain stability, rendering it less suitable for patients with poor bone quality, advanced spondylosis, or multi-level disc pathology. Older patients, who are more likely to present with comorbidities such as diabetes mellitus, hypertension, and dyslipidemia, often derive greater benefit from fusion procedures, which provide definitive stabilization of diseased segments [20,31]. These relative indications and contraindications help explain the higher prevalence of ACDF among older individuals with myelopathy and advanced disc degeneration in our study population.

Overall, CDA demonstrated complication rates comparable to or lower than ACDF, with overall incidences of 5.81% and 7.29%, respectively. ACDF was associated with a higher prevalence of dysphagia (4.90% vs. 3.60%) and a greater need for perioperative blood transfusion (0.13% vs. 0.00%). Conversely, CDA patients experienced slightly higher rates of cervical spinal cord injury (0.30% vs. 0.17%) and urinary tract infections. The existing literature presents mixed findings: several studies have reported fewer adverse events following CDA [32,33], whereas others have noted either no difference or higher complication rates compared with ACDF [34,35]. It is important to note that the NIS database is limited to ICD-10 codes entered by treating providers and does not contain operative details. Therefore, these associations should be interpreted with caution and cannot be regarded as definitive causal explanations. Given the similarities in surgical approach, both procedures demonstrated comparable rates of approach-related complications. While the large sample size of the NIS database allowed us to detect statistically significant differences between ACDF and CDA, several of these differences are clinically modest and are unlikely to influence clinical decision-making on an individual patient level. These findings underscore the importance of distinguishing between statistical and clinical significance when interpreting large administrative datasets.

A recent systematic review and meta-analysis found that CDA was associated with a significantly lower incidence of secondary surgeries and adverse events compared to ACDF, without significant differences in neurological success [36]. Similarly, another study reported no statistically significant differences in the incidence of spinal cord injury or other major complications between the two techniques [37].

Although the present analysis identified statistically significant differences in certain complication rates between CDA and ACDF, the absolute rates were low for both procedures. These findings suggest that while statistical differences exist, their clinical impact may be limited. Future research should aim to determine whether these differences translate into meaningful variations in long-term patient outcomes, healthcare utilization, and quality of life.

In this study, a significantly greater proportion of CDA patients (64.9%) were covered by private insurance compared with ACDF patients (44.5%). Similar trends have been reported in previous analyses. For example, a study utilizing the National Inpatient Sample (NIS) from 2006 to 2013 found that 66.2% of CDA patients had private insurance versus 55.4% of ACDF patients [19]. This disparity likely reflects differences in patient age and eligibility, as younger individuals—who are more likely to have private insurance—are also more likely to meet selection criteria for CDA. In contrast, older patients, particularly those covered by Medicare, may be less frequently considered for arthroplasty due to the presence of advanced degenerative changes or other contraindications. Additionally, variation in insurance coverage policies, including reimbursement rates and authorization practices, may contribute to the observed differences in payer distribution.

In terms of resource utilization, CDA patients in our cohort had a modestly shorter mean length of stay compared with ACDF patients (1.32 vs. 1.39 days) yet incurred substantially higher total hospital charges (USD 82,431 vs. USD 58,472). These findings are consistent with prior studies [19,38] that also demonstrated reduced length of stay for CDA relative to ACDF, while highlighting the potential impact of device costs, surgical instrumentation, and reimbursement structures on total expenditures.

The higher costs associated with CDA compared with ACDF are primarily attributable to the increased expense of implants and surgical instrumentation required for arthroplasty. CDA implants, incorporating motion-preserving technology, are generally more costly than the devices used in ACDF [39,40]. A study published in World Neurosurgery reported that the mean supply cost for CDA was approximately USD 9532, compared to USD 4173 for ACDF, with the majority of this discrepancy attributable to the higher price of disc replacement implants [39]. Beyond implant costs, total intraoperative expenses are also greater for CDA; the same study found mean intraoperative costs of USD 12,026 for CDA versus USD 6776 for ACDF. This difference reflects not only the increased cost of implants but also the additional operative time and resources required for arthroplasty [40].

Economic trends further highlight the growing financial burden associated with CDA. Between 2009 and 2019, the mean total hospital charges for elective CDA increased by 73%, while the mean total cost for index hospital admissions rose by 26% [40]. Notably, this cost escalation has not been matched by a proportional rise in reimbursements, resulting in higher out-of-pocket expenses for patients and increased financial strain on healthcare systems. These findings underscore the importance of considering the long-term economic implications of CDA relative to ACDF when evaluating its broader adoption in clinical practice.

The shorter length of stay (LOS) observed in CDA patients may reflect more standardized perioperative care protocols and implant-specific surgical workflows. In contrast, the higher costs associated with CDA are likely attributable to the advanced technology, specialized implants, and longer operative times required for these procedures. In resource-limited settings, such elevated costs can place substantial strain on healthcare budgets, potentially restricting the availability of CDA. Consequently, ACDF is often favored in such environments, as it demonstrates greater cost-effectiveness across various willingness-to-pay thresholds [41]. The financial burden associated with CDA may also disproportionately limit access for patients from lower socioeconomic backgrounds or those without comprehensive insurance coverage [12].

Accurate determination of indications and patient eligibility is essential when selecting between CDA and ACDF. Inappropriate selection for CDA can compromise surgical outcomes. For example, if the posterior longitudinal ligament is divided during the removal of posterior osteophytes, segmental fusion is generally preferred over disc replacement to avoid iatrogenic instability [42].

CDA is most commonly indicated for patients with single- or two-level cervical disc disease between the C3 and C7 levels. Regulatory approval for CDA is based on clinical trials demonstrating non-inferiority to ACDF, with evidence indicating that, in appropriately selected patients, CDA can provide comparable or superior clinical and functional outcomes while preserving segmental motion [43,44]. Proper patient selection is critical, with absolute contraindications including severe osteoporosis, active infection, and significant cervical instability due to the heightened risk of implant failure and poor postoperative outcomes. Relative contraindications—such as segmental kyphosis or prior cervical spine surgery—require individualized assessment, as emerging evidence suggests that CDA may remain a viable option in select patients with outcomes comparable to standard candidates [45,46]. While preoperative segmental mobility has historically been regarded as a key criterion for CDA candidacy, recent studies suggest that even patients with reduced baseline mobility can achieve meaningful postoperative improvements in pain relief and functional status, challenging the traditional reliance on mobility as a strict determinant [47].

Over the long term, outcomes tend to be similar—and in some cases slightly better—with CDA, largely due to fewer reoperations and less adjacent segment degeneration. These advantages suggest that the higher upfront costs of CDA may balance out over time [48,49]. That said, the greatest cost-effectiveness has been reported particularly in patients with two-level disease and in health systems where time away from work and productivity loss play a major role in overall costs [50].

This study acknowledges several limitations inherent to its methodological approach, which is based on the use of a broad set of ICD-10 procedure and diagnosis codes applied to a large administrative dataset. While this strategy enables a macro-level assessment of national trends and facilitates the analysis of a substantial sample size—approximately 98,000 single-level CDA and ACDF cases—it does not permit granular, patient-level clinical detail. This reflects an inherent trade-off between the depth of individual patient information and the statistical power afforded by large-scale, population-based analyses. Additionally, the cost estimates reported in the National Inpatient Sample (NIS) are derived from hospital-specific cost-to-charge ratios, which may overestimate actual procedural expenses. However, these ratios undergo internal validation by the Agency for Healthcare Research and Quality, supporting their use in comparative economic analyses. Despite the use of validated ICD-10 procedure and diagnosis codes to optimize accuracy, the reliance on administrative coding within the NIS introduces the potential for misclassification or miscoding [51]. Coding inaccuracies may occur at the hospital level due to human error or variations in documentation practices, and these errors could influence the precision of reported outcomes. While such limitations are inherent to large administrative databases and are unlikely to systematically bias comparisons between study cohorts, they should be considered when interpreting the findings.

## 5. Conclusions

In conclusion, this study demonstrates a clear and sustained increase in the utilization of CDA, particularly among younger patients and those with private insurance coverage. CDA was associated with shorter hospital stays and lower rates of inpatient complications, although at the expense of higher hospitalization costs.

## Figures and Tables

**Table 1:** ICD-10 and procedure codes used for case selection and variable definition.

Category	ICD 10 CODES
Cervical Disc Arthroplasty (CDA)	0RR30JZ, 0RR20JZ
Anterior Cervical Discectomy and Fusion (ACDF)	0RG10A0, 0RG10A1, 0RG10A4, 0RG10J0, 0RG10J1, 0RG10J4
Heart Failure	I5021, I5031, I5033, I5041, I5043
Acute Kidney Injury	N170, N171, N172, N178, N179
Acute Coronary Artery Disease	I2101, I2102, I2109, I211, I2119, I2111, I212, I2129, I213, I214, I219
Stroke	I60, I61, I62, I63, I650, I688, O873, O2250, O2251, O2252
Pulmonary Edema	J810, J811, I501
Hypertension	I10(start with)
Blood Loss Anemia	D62 (start with)
Pneumonia	J189, J159, J22
Pulmonary Embolism	I2602, I2609, I2692, I2699
DVT	I82401, I82402, I82403, I82409, I82411, I82412, I82413, I82419, I82421, I82422, I82423, I82429
Dyslipidemia	E78 (start with)
Obstructive Sleep Apnea	G473
Chronic Anemia	D64 (start with)
Alcohol Abuse History	F10
Osteoporosis	M81, M82
Mental Disorders	F (start with)
Parkinson’s Disease	G20 (start with)
Type 2 Diabetes Mellitus	E11 (start with)
Chronic Kidney Disease	N18 (start with)
Congestive Heart Failure	I500, I501, I509
Chronic Lung Disease	J44 (start with)
History of Myocardial Infarction	I252
Peripheral Vascular Disease	I73 (start with)
History of Cerebrovascular Accident (CVA)	Z8673, I69 (start with)
Dementia	F03 (start with)
Peptic Ulcer Disease	K25-K28
Hemiplegia	G81
Neoplasms	C (start with)
Neoplasms of Lymphoid and Hematopoietic Tissue	C81-C96

**Table 2:** Demographic and payer characteristics of patients undergoing ACDF and CDA.

Parameter	ACDF	CDA	Significance
Total Surgeries (%)	85,584	11,415	-
Average Age (y)	55.6	47.2	*p* < 0.001
Female (%)	51.7	52.5	*p* = 0.108
Primary expected payer—Medicare (%)	33.9	10.7	*p* < 0.001
Primary expected payer—Medicaid (%)	10.7	9.7	
Primary expected payer—private including HMO (%)	44.5	64.9	
Primary expected payer—self-pay (%)	1.2	1.3	
Primary expected payer—no charge (%)	0.1	0	
Primary expected payer—other (%)	9.6	13.3	

**Table 3:** Prevalence of comorbidities among patients undergoing ACDF and CDA.

Parameter	ACDF (*n* = 85,584)	CDA (*n* = 11,415)	Significance
Hypertension (%)	43.7	25.1	*p* < 0.001
Dyslipidemia (%)	30	17.3	*p* < 0.001
Obstructive Sleep Apnea (%)	9.5	6.9	*p* < 0.001
Chronic Anemia (%)	2.3	1.8	*p* < 0.001
Alcohol Abuse (%)	1.2	0.8	*p* < 0.001
Osteoporosis (%)	2.3	0.9	*p* < 0.001
Parkinson Disease (%)	0.5	0.1	*p* < 0.001
Alzheimer Disease (%)	0.1	0	*p* = 0.698
Chronic Kidney Disease (%)	3.8	1.1	*p* < 0.001
Congestive Heart Failure (%)	0.9	0.1	*p* < 0.001
Chronic Lung Disease (%)	8	3.2	*p* < 0.001
Diabetes Mellitus (%)	19.5	9.6	*p* < 0.001

The continuation of this table is provided in the Table S1.

**Table 4:** Comparison of demographic and clinical characteristics in propensity score-matched cohorts undergoing ACDF and CDA.

Parameter	ACDF (*n* = 11,415)	CDA (*n* = 11,415)	Significance
Average Age (y)	47.3	47.2	*p* = 0.36
Female (%)	52.1	52.5	*p* = 0.62
Primary expected payer—Medicare (%)	11.6	11.3	*p* = 0.41
Primary expected payer—Medicaid (%)	9.7	9.7	
Primary expected payer—private including HMO (%)	64.5	64.9	
Primary expected payer—self-pay (%)	1.5	1.3	
Primary expected payer—no charge (%)	0	0	
Primary expected payer—other (%)	12.7	12.7	
Hypertension (%)	24.7	25.1	*p* = 0.59
Dyslipidemia (%)	17.2	17.3	*p* = 0.93
Obstructive Sleep Apnea (%)	6.4	6.9	*p* = 0.05
Chronic Anemia (%)	1.6	1.8	*p* = 0.43
Osteoporosis (%)	0.9	0.9	*p* = 0.52
Chronic Kidney Disease (%)	1	1.1	*p* = 0.51
Chronic Lung Disease (%)	2.9	3.2	*p* = 0.09
Diabetes Mellitus (%)	8.9	9.6	*p* = 0.05
Obesity (%)	16.4	15.6	*p* = 0.09

The continuation of this table is provided in the Table S2.

**Table 5:** Comparison of hospitalization outcomes in propensity score-matched cohorts undergoing ACDF and CDA.

	ACDF (*n* = 11,415)	CDA (*n* = 11,415)	Significance
**Length of stay mean in days**	1.39 (Std. deviation 1.52)	1.32 (Std. deviation 1.27)	*p* < 0.001
**Total charges mean in USD**	58,472 (Std. deviation 41703)	82,431 (Std. deviation 53105)	*p* < 0.001

**Table 6:** Postoperative outcomes in patients undergoing ACDF and CDA after propensity score matching.

Parameter	ACDF (*n* = 11,415)	CDA (*n* = 11,415)	Significance	Odds Ratio	Odds Ratio 95% Confidence
Dysphagia (%)	4.90%	3.60%	*p* < 0.001	0.724	0.63–0.82
Cervical spinal cord injury (%)	0.17%	0.30%	*p* = 0.04	1.752	1.01–3.03
UTI (%)	0.22%	0.39%	*p* = 0.02	1.803	1.1–2.94
Blood transfusion (%)	0.13%	0.00%	*p* = 0.01	0.500	0.17–1.46
Venous Thromboembolism (%)	0.13%	0.04%	*p* = 0.03	0.308	0.12–0.75
Ileus (%)	0.08%	0.17%	*p* = 0.07	2.002	0.93–4.27
Feeding Tube (%)	0.08%	0.00%	*p* = 0.01	0.5	0.17–1.46
Dural tear (%)	0.04%	0.04%	*p* = 1.00	1.000	0.28–3.45
Pulmonary Embolism (%)	0.04%	0.00%	*p* = 0.03	0.500	0.49–0.5
Mortality (%)	0.00%	0.00%	*p* = 1.00	-	

The continuation of this table is provided in the Table S3.

## Data Availability

Restrictions apply to the availability of these data. Data were obtained from HCUP and are available [https://hcup-us.ahrq.gov/] with the permission of HCUP.

## Supplementary Materials

The following supporting information can be downloaded at https://www.mdpi.com/article/10.3390/jcm14186559/s1, Table S1: Prevalence of Comorbidities Among Patients Undergoing ACDF and CDA; Table S2: Comparison of Demographic and Clinical Characteristics in Propensity Score-Matched Cohorts Undergoing ACDF and CDA; Table S3: Postoperative Outcomes in Patients Undergoing ACDF and CDA After Propensity Score Matching.

## Abbreviations

ACDF	Anterior Cervical Decompression and Fusion
CDA	Cervical Disc Arthroplasty
HCUP	Healthcare Cost and Utilization Project
ICD-10	International Classification of Diseases, 10th Revision
NIS	Nationwide Inpatient Sample
SPSS	Statistical Package for the Social Sciences
