# Supplementary material for: Comparative Analysis of Cervical Disc Arthroplasty and Anterior Cervical Discectomy and Fusion: Trends, Demographics, and Clinical Outcomes in a Nationwide Inpatient Sample
Source: J Clin Med. 2025 Sep 18;14(18):6559. doi: 10.3390/jcm14186559 (PMC12470919; doi:10.3390/jcm14186559)
Supplement: Supplementary file 1 [file jcm-14-06559-s001.zip › jcm-3840338-supplementary.pdf]

Supplementary tables:

Table S1: Prevalence of Comorbidities Among Patients Undergoing ACDF and CDA.

Parameter	ACDF (n=85,584)	CDA (n=11,415)	Significance
Hypertension (%)	43.7	25.1	P<0.001
Dyslipidemia (%)	30	17.3	P<0.001
Obstructive Sleep Apnea (%)	9.5	6.9	P<0.001
Chronic Anemia (%)	2.3	1.8	P<0.001
Alcohol Abuse (%)	1.2	0.8	P<0.001
Osteoporosis (%)	2.3	0.9	P<0.001
Parkinson Disease (%)	0.5	0.1	P<0.001
Alzheimer Disease (%)	0.1	0	P=0.698
Chronic Kidney Disease (%)	3.8	1.1	P<0.001
Congestive Heart Failure (%)	0.9	0.1	P<0.001
Chronic Lung Disease (%)	8	3.2	P<0.001
Diabetes Mellitus (%)	19.5	9.6	P<0.001

IBD (%)	0.5	0.3	P<0.001
Liver Disease (%)	1.1	0.7	P<0.001
Obesity (%)	18.5	15.6	P<0.001
Fibromyalgia (%)	3.8	3	P<0.001
Disorders of Thyroid (%)	11.9	9.4	P<0.001
History of Myocardial Infarction (%)	2.9	0.7	P<0.001
Peripheral Vascular Disease (%)	1.3	0.6	P<0.001
History of Cerebrovascular Accident (%)	3.9	1.3	P<0.001
Dementia (%)	0.2	0.2	P<0.001
Neoplasms (%)	0.8	0.3	P<0.001
Neoplasms of Lymphoid and Hematopoietic Tissue (%)	0.3	0.1	P<0.001

Table S2: Comparison of Demographic and Clinical Characteristics in Propensity Score-Matched Cohorts Undergoing ACDF and CDA.

Parameter	ACDF (n=11,415)	CDA (n=11,415)	Significance
Average Age (y)	47.3	47.2	P=0.36
Female (%)	52.1	52.5	P=0.62
Primary expected payer - Medicare (%)	11.6	11.3	P=0.41
Primary expected payer - Medicaid (%)	9.7	9.7	
Primary expected payer - private including HMO (%)	64.5	64.9	
Primary expected payer - self-pay (%)	1.5	1.3	
Primary expected payer - no charge (%)	0	0	
Primary expected payer - other (%)	12.7	12.7	
Hypertension (%)	24.7	25.1	P=0.59
Dyslipidemia (%)	17.2	17.3	P=0.93
Obstructive Sleep Apnea (%)	6.4	6.9	P=0.05
Chronic Anemia (%)	1.6	1.8	P=0.43

Alcohol Abuse (%)	0.7	0.8	P=0.33
Osteoporosis (%)	0.9	0.9	P=0.52
Parkinson Disease (%)	0	0.1	P=0.20
Alzheimer Disease (%)	0	0	P=1
Chronic Kidney Disease (%)	1	1.1	P=0.51
Congestive Heart Failure (%)	0.1	0	P=0.06
Chronic Lung Disease (%)	2.9	3.2	P=0.09
Diabetes Mellitus (%)	8.9	9.6	P=0.05
Inflammatory Bowel Disease (%)	0.4	0.3	P=0.23
Liver Disease (%)	0.5	0.7	P=0.19
Obesity (%)	16.4	15.6	P=0.09
Fibromyalgia (%)	2.7	3	P=0.11
History of Myocardial Infarction (%)	0.6	0.6	P=0.18
Peripheral Vascular Disease (%)	0.6	0.6	P=0.35
History of Cerebrovascular Accident (%)	1.3	1.3	P=0.42
Neoplasms (%)	0.4	0.3	P=0.09

Neoplasms of Lymphoid and Hematopoietic Tissue (%)	0.1	0.1	P=1

Table S3: Postoperative Outcomes in Patients Undergoing ACDF and CDA After Propensity Score Matching

Parameter	ACDF (n=11,415)	CDA (n=11,415)	Significance	Odds Ratio	Odds Ratio 95% Confidence
Dysphagia (%)	4.90%	3.60%	P<0.001	0.724	0.63 - 0.82
Blood Loss Anemia (%)	1.00%	0.80%	P=0.17	0.825	0.62 - 1.08
Cervical spinal cord injury (%)	0.17%	0.30%	P=0.04	1.752	1.01 - 3.03
UTI (%)	0.22%	0.39%	P=0.02	1.803	1.1 - 2.94
Acute Renal Failure (%)	0.21%	0.26%	P=0.50	1.201	0.7 - 2.04
Pneumonia (%)	0.17%	0.17%	P=1.00	1.000	0.53 - 1.86
Blood transfusion (%)	0.13%	0.00%	P=0.01	0.500	0.17 - 1.46
Venous Thromboembolism (%)	0.13%	0.04%	P=0.03	0.308	0.12 - 0.75
Pulmonary Edema (%)	0.04%	0.04%	P=1.00	1.000	0.28 - 3.45
Ileus (%)	0.08%	0.17%	P=0.07	2.002	0.93 - 4.27
Feeding Tube (%)	0.08%	0.00%	P=0.01	0.5	0.17 - 1.46
Dural tear (%)	0.04%	0.04%	P=1.00	1.000	0.28 - 3.45
Sepsis (%)	0.04%	0.00%	P=0.03	0.500	0.49 - 0.5
Pulmonary Embolism (%)	0.04%	0.00%	P=0.03	0.500	0.49 - 0.5
Mortality (%)	0.00%	0.00%	P=1.00	-	
